# CYP3A5*3 and CYP3A4*22 Cluster Polymorphism Effects on LCP-Tac Tacrolimus Exposure: Population Pharmacokinetic Approach

**DOI:** 10.3390/pharmaceutics15122699

**Published:** 2023-11-29

**Authors:** Zeyar Mohammed Ali, Marinda Meertens, Beatriz Fernández, Pere Fontova, Anna Vidal-Alabró, Raul Rigo-Bonnin, Edoardo Melilli, Josep M. Cruzado, Josep M. Grinyó, Helena Colom, Nuria Lloberas

**Affiliations:** 1 Nephrology Department, Hospital Universitari de Bellvitge-IDIBELL, 08908 Barcelona, Spain; zraoofmo18@alumnes.ub.edu (Z.M.A.); marindameertens@hotmail.com (M.M.); bea.fernandez.a@gmail.com (B.F.); perefontova@ub.edu (P.F.); avidala@idibell.cat (A.V.-A.); emelilli@bellvitgehospital.cat (E.M.); jmcruzado@bellvitgehospital.cat (J.M.C.); 2 Biopharmaceutics and Pharmacokinetics Unit, Department of Pharmacy and Pharmaceutical Technology and Physical Chemistry, School of Pharmacy, University of Barcelona, 08007 Barcelona, Spain; 3 Biochemistry Department, Hospital Universitari de Bellvitge-IDIBELL, 08908 Barcelona, Spain; raulr@bellvitgehospital.cat; 4 Department of Clinical Sciences, Medicine Unit, University of Barcelona, 08007 Barcelona, Spain; jgrinyo@ub.edu

**Keywords:** Tacrolimus, LCP-Tac, population pharmacokinetics, CYP3A5, CYP3A4, ABCB1, renal transplantation, stable adult patients, immunosuppression

## Abstract

The aim of the study is to develop a population pharmacokinetic (PopPK) model and to investigate the influence of CYP3A5/CYP3A4 and ABCB1 single nucleotide polymorphisms (SNPs) on the Tacrolimus PK parameters after LCP-Tac formulation in stable adult renal transplant patients. The model was developed, using NONMEM v7.5, from full PK profiles from a clinical study (n = 30) and trough concentrations (C_0_) from patient follow-up (n = 68). The PK profile of the LCP-Tac formulation was best described by a two-compartment model with linear elimination, parameterized in elimination (CL/F) and distributional (CL_D_/F) clearances and central compartment (Vc/F) and peripheral compartment (Vp/F) distribution volumes. A time-lagged first-order absorption process was characterized using transit compartment models. According to the structural part of the base model, the LCP-Tac showed an absorption profile characterized by two transit compartments and a mean transit time of 3.02 h. Inter-individual variability was associated with CL/F, Vc/F, and Vp/F. Adding inter-occasion variability (IOV) on CL/F caused a statistically significant reduction in the model minimum objective function MOFV (*p* < 0.001). Genetic polymorphism of CYP3A5 and a cluster of CYP3A4/A5 SNPs statistically significantly influenced Tac CL/F. In conclusion, a PopPK model was successfully developed for LCP-Tac formulation in stable renal transplant patients. CYP3A4/A5 SNPs as a combined cluster including three different phenotypes (high, intermediate, and poor metabolizers) was the most powerful covariate to describe part of the inter-individual variability associated with apparent elimination clearance. Considering this covariate in the initial dose estimation and during the therapeutic drug monitoring (TDM) would probably optimize Tac exposure attainments.

## 1. Introduction

Tacrolimus (Tac) is a major immunosuppressant drug prescribed for over 30 years in kidney transplant patients to avoid graft rejection [1,2,3]. High inter- and intra-individual variabilities in Tac exposure have been reported [4,5]. Among the factors, there are hematocrit levels, time after transplantation, hepatic dysfunction, protein plasma concentrations, as well as patients’ age, ethnicity, sex, and CYP3A4/A5 polymorphisms (SNPs), which have been described as being mainly responsible [4,6,7,8,9,10,11]. These factors and the Tac narrow therapeutic index are challenging for the establishment of an optimal dose. Indeed, Tac might be associated with several dose-dependent side effects such as neurotoxicity, nephrotoxicity, and post-transplant diabetes mellitus [12,13,14], which can be reduced by avoiding a Tac pre-dose concentration (C_0_), too high in the long-term. Conversely, Tac under-dosing, resulting in too-low C_0_, should not be attempted because it increases the risk of acute rejection and immunologic sensitization [15]. In this regard, the dosage of the Tac formulation can play an important role. Several formulations of Tac have been developed, demonstrating a very high efficacy in preventing an acute rejection episode. Thus, Tac was originally formulated as an immediate-release IR-Tac (Prograf^®^, Astellas Pharma Europe Ltd., Staines, UK), administered twice daily. Then, the first once-daily prolonged-release formulation, (Advagraf^®^, Astellas Pharma Europe Ltd., Staines, UK) was developed, and lastly, the once-daily extended-release formulation LCP-Tac was commercialized using a MeltDose^®^ delivery technology that provides a slower release/absorption rate, resulting in delayed Tmax and more decreased Cmax and fluctuation profile. Additionally, compared to the IR-Tac, reduction in Tac to the smallest possible particle size in LCP-Tac allows a better dissolution and absorption, increasing its bioavailability [16,17,18,19,20]. Other factors, such as the varying distribution of CYP3A enzymes along the gut, may contribute to the increasing bioavailability of LCP-Tac compared to IR-Tac [21]. Part of the Tac from the LCP-Tac formulation is absorbed in distal parts of the small intestine or even the colon, where the CYP3A enzyme exists to a lesser extent compared to the proximal parts, where most of the IR-Tac is absorbed.

The exposure and starting dose of Tac is significantly influenced by gene-encoding Tac-metabolizing enzyme cytochromes P450 3A4/5 (CYP3A4/5). CYP3A5 expresser (*1 allele carrier) requires higher Tac dose in comparison to non-expresser (*3/*3) to achieve similar exposure [22,23,24,25]. It is suggested that a lower dose is required for patients with CYP3A4*22 SNP [26,27], as it leads to lower Tac oral clearance. In addition, ABCB1 is also thought to be a contributing factor to the low oral bioavailability of Tac. However, the impact ABCB1 SNPs on Tac pharmacokinetics has not been conclusively determined [28].

The influence of the predictive factors of Tac exposure widely studied after IR-Tac administration using modeling approaches [4,29,30,31,32,33,34] has been widely studied. Although only a few studies describe the influence of these factors for LCP-Tac, of these, only two have been carried out in kidney transplant patients [35,36,37]. This study aims at investigating the influence of CYP3A5, CYP3A4, and ABCB1 SNPs on the Tac exposure upon LCP-Tac administration, in stable adult renal transplant patients, using a population-modeling approach.

## 2. Methods

### 2.1. Study Design

Thirty patients were selected from an open-label, prospective, non-randomized investigator-driven single-center clinical trial (clinicalTrials.gov NCT02961608), whereas the rest of the patients were taken from routine check-ups at the hospital with Tac C_0_. All the patients were treated with an immunosuppressive drug regimen of oral twice-daily IR-Tac (Prograf; Astellas Pharma Europe Ltd., Staines, UK), for at least six months before conversion to a once-daily LCP-Tac oral regime (Envarsus; Chiesi Farmaceutici, Parma, Italy) with a dose conversion ratio of 0.7. All patients received triple immunosuppression therapy combining Tac, mycophenolate mofetil, and prednisone.

A total of 98 stable renal transplant patients who had undergone a kidney transplant at least six months prior to the study were included. Patients with current infections, hepatitis B or C, severe gastrointestinal disorders, and patients receiving concomitant drugs that could interact with the CYP3A enzyme were excluded from this study. The study was conducted in compliance with the Declaration of Helsinki and approved by the ethics committee of the Bellvitge University Hospital (Barcelona, Spain).

Patients from the clinical trial had an extensive sampling profile (10–18 sampling times over a 24 h period). Blood samples were taken at pre-dose, 0.5, 1, 1.5, 2, 3, 4, 6, 8, 12, 12.5, 13, 13.5, 14, 15, 20, and 24 h post-dosing under steady-state conditions. For the remaining patients, from one to five C_0_ samples per patient were obtained, depending on the date of conversion.

### 2.2. Tacrolimus Measurement and Data Recording

Tac was measured using an LC-MS/MS method, previously developed and validated [38]. Chromatographic determination was performed using the Acquity (^®^) UPLC (^®^) with a C18 BEH ™ reversed-phase column (2.1 × 50 mm id, 1. 7 μm). The limit of quantitation was set at 1.0 ng·mL^−1^. At the start of the treatment, Tac daily doses and demographic characteristics of the patients were retrieved from the medical files. For hematocrit (%) and serum creatinine concentrations (µmol·L^−1^), their concentrations on each occasion were also monitored and recorded. The clinical outcome variables that were assessed were renal function (eGFR), estimated using the Chronic Kidney Disease Epidemiology Collaboration formula, delayed graft function (DGF), and graft loss.

### 2.3. Genotyping

Genomic DNA was extracted from a peripheral whole-blood sample using Maxwell RSC^®^ (Promega Corporation, Sydney, Australia) and was stored at −80 °C. Genotyping of the CYP3A5*3 G > A (rs776746), CYP3A4*22 C > T (rs35599367), and ABCB1 3435C > T (rs1045642) polymorphisms (SNPs) was carried out using TaqMan SNP Genotyping Assay with the 7900HT Fast Real-time PCR System, Applied Biosystems (Thermo Fisher Scientific, Waltham, MA, USA).

According to the functional defect associated with CYP3A variants, we classified patients into three different clusters of CYP3A metabolizers: poor metabolizers (PM) (CYP3A4*22 carriers + CYP3A5*3/*3), intermediate metabolizers (IM) (CYP3A4*22 non-carriers + CYP3A5*3/*3 or CYP3A4*22 carriers + CYP3A5*1 carriers), and high metabolizers (HM) (CYP3A4*22 non-carriers + CYP3A5*1 carriers).

### 2.4. Statistical Analysis

Unpaired *t*-tests were used to evaluate differences in log-transformed values of exposure metrics (C_0_ normalized by dose and AUC concentration-time curves) between two groups, and at the same time, one-way analysis of variance tests were used for comparisons among three the groups. Results with *p* values of less than 0.05 were considered statistically significant. Statistical analyses were performed with the R program version (4.0.3).

### 2.5. Population Pharmacokinetic Analysis

Base Model Development

The popPK analysis was performed with the non-linear mixed-effects model approach using the NONMEM^®^ version 7.5 (ICON Development Solutions, Hanover, MD, USA). Perl-Speaks-NONMEM (PsN) version 5.2.6, R package version 4.0.3, Pirana Modeling Workbench version 3.0 (Certara L.P. (Pharsight), St. Louis, MO, USA), and Xpose 4.7.2 were used as support tools for model evaluation. The first-order conditional estimation (FOCEI) method with interaction was used throughout the modeling process.

One- and two-compartment open models with linear elimination and first-order absorption were tested. The standard lag model and transit compartment models, in which the optimal amount of transit compartments is estimated [39], were used to model the absorption delay. Inter-individual variability (IIV) and inter-occasion variability (IOV) [40] were tested in all PK parameters assuming log-normal distributions. Additive, proportional, and combined error models were tested to describe the residual error (RE) variability. To compare the different tested models statistically, the likelihood ratio test, based on the reduction in the minimum objective function value (MOFV) at a significance level of *p* < 0.005 (change in MOFV [∆MOFV] = –7.879 for 1 degree of freedom) was considered. For non-hierarchical models, the most parsimonious model according to the Akaike information criterion (AIC) was chosen [41]. The decrease in MOFV, parameter precision expressed as a percentage of relative standard error (RSE%), reductions in IIV associated with parameters, η- and ε-shrinkage values [42], model completion status, and visual inspection of goodness-of-fit plots were also considered for model selection.

Covariate Model

The effect of factors that could be physiologically and clinically meaningful on the PK parameters was investigated. Specifically, the influence of patient age, gender, total body weight, body mass index, and hematocrit were evaluated. The effects of CYP3A4/A5 SNPs, cluster phenotypes, and ABCB1 genotypes were also tested on Tac CL/F.

Firstly, correlations between continuous covariates were explored. Then, univariate analysis, forward inclusion, and backward elimination stepwise procedures were carried out to explore the covariates [43]. Significance levels of 5% (ΔMOFV = –3.841 units, *p* < 0.05) and 0.1% (ΔMOFV = 10.8 units, *p* < 0.001) were considered during the forward addition and backward elimination steps, respectively. Only covariates providing a reduction in IIV associated with parameters of at least 10% were considered clinically relevant and were retained in the model.

### 2.6. Model Evaluation and Internal Validation

Goodness-of-fit plots were analyzed throughout the modeling process. The predictive capability was evaluated using prediction-corrected visual predictive checks (pcVPC) based on 1000 simulations [44]. The median and 2.5th and 97.5th percentiles of the simulated data and their respective 95% prediction intervals were calculated and visually compared with the same percentiles obtained from the original raw data. Also, Npde [45] (normalized prediction distribution errors) diagnostics were performed. Model adequacy was evaluated by checking the even distribution of predicted discrepancies and comparing the shape, location, and variance of distribution parameters to the theoretical normal distribution. 

## 3. Results

### 3.1. Patient Characteristics and Datasets

Blood Tac concentration-time data (n = 655) from 98 renal transplant recipients were simultaneously analyzed. From this data, 480 out of 655 were obtained from the rich AUC sampled group (n = 30), at least six months after transplantation, and 175 were obtained from C_0_ values (n = 68).

**Figure 1:**
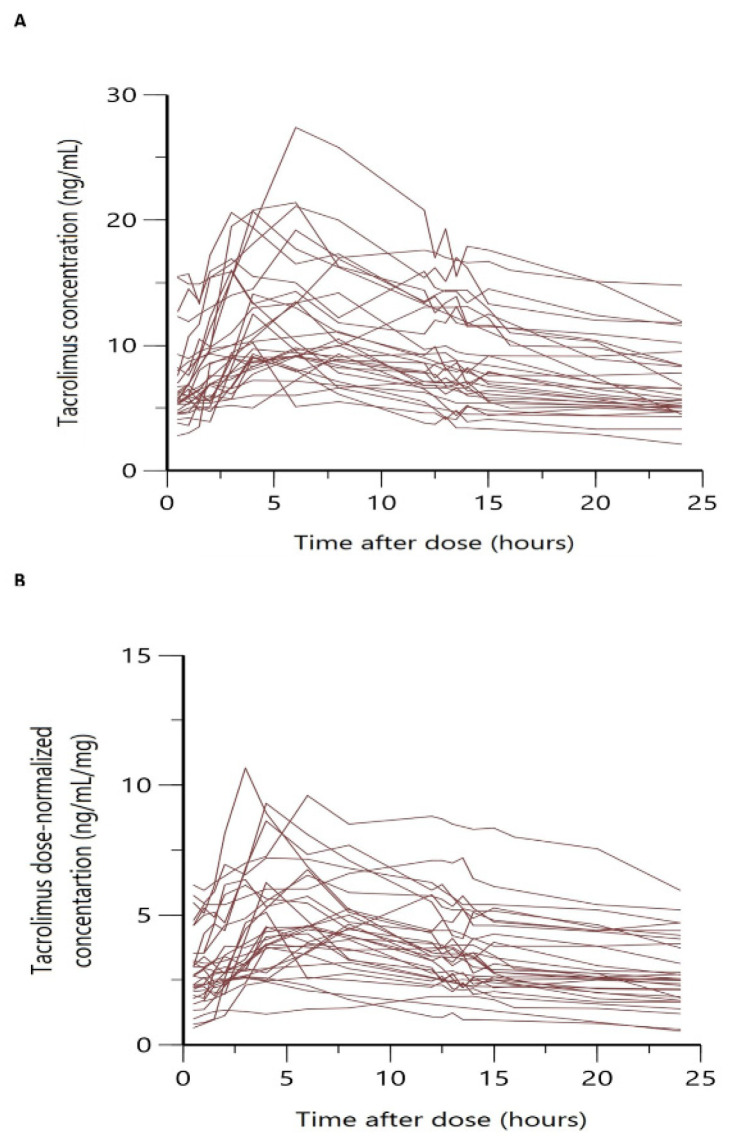
(**A**) Overlaid individual steady-state PK profiles of Tac following LCP-Tac administration. (**B**) Overlaid normalized by dose individual steady-state PK profiles of Tac following LCP-Tac administration.

The median total daily administered dose was 2 mg with a wide variation between patients ranging from 0.5 mg to 12 mg. Figure 1 shows the overlaid individual full concentration time and normalized-by-dose concentration time profiles. In both cases, a large variability was observed, especially in the absorption phase, with a wide range of variation in Tmax among patients and more than one peak in some cases.

Demographic, biochemical, and genetic characteristics of the patient population are summarized in Table 1. Patient characteristics were similar when both groups were compared (clinical and follow-up study). Similar values in the hematocrit, serum creatinine, and renal function values between groups were observed. Table 2 displays comparative C_0_ and AUC values sorted by the CYP3A4/A5, ABCB1 SNPs groups, and cluster phenotypes (Table 2). Statistically significant (*p* < 0.001) differences were found when comparing normalized by dose AUC values between CYP3A5 *1 expressers vs. non-expressers. Similarly, statistically significant (*p* < 0.001) differences were found when comparing C_0_ normalized by a dose of CYP3A5 *1 expressers vs. non-expressers.

Regarding CYP3A4, no significant difference (*p* > 0.05) was found when comparing the normalized-by-dose AUC values between CYP3A4*22 carriers vs. CYP3A4*22 non-carriers. In contrast, CYP3A4*22 carriers vs. CYP3A4*22 non-carriers C_0_ normalized by dose showed significant differences when compared (*p* < 0.01).

When cluster combinations were considered, the C_0_ normalized by dose was significantly different among all phenotypes (*p* < 0.01) (Table 2). For AUC/D values, statistically significant differences were found among all phenotypes, except between IM and PM (Table 2).

In contrast, no statistically significant differences were found when AUC values were compared; that is, the normalized-by-dose ABCB1 *C carriers (high pumper) vs. non-carriers (low pumper) (*p* > 0.05).

### 3.2. Population PK Analysis

The PK profile of the Tac whole-blood concentration versus time data was best described by a two-compartment model with linear elimination, parameterized in terms of apparent elimination and distributional clearances (CL/F and CL_D_/F, respectively) and apparent central compartment (V_c_/F) and peripheral compartment (V_p_/F) distribution volumes. A time-lagged first-order absorption process was characterized using transit compartment models and the number of absorption compartments were fixed to 2. According to the base model, the MeltDose^®^ Tac shows a mean absorption transit time of 2 h 55 min. Inter-individual variability could be associated with CL/F, V_c_/F, and V_p_/F. Adding IOV on CL/F caused a statistically significant reduction in the model MOFV (∆MOFV = −232 units, *p* < 0.005) and was therefore retained in the final model. Further, inclusion of the IOV on other parameters did not improve the model. A proportional error model best described the RE distribution. When covariates were entered univariately on distribution Vc/F and V_p_/F volumes or clearances, neither body weight nor body mass index or age provided a significant drop in the MOFV (*p* > 0.05). Allometric inclusion of body weight on these PK parameters worsened the model. No improvement of the model was observed when residual error associated with concentrations was standardized by hematocrit values of 45%.

Genetic polymorphism in CYP3A5 (*1 expressers vs. non-expressers) statistically significantly influenced Tac CL/F, resulting in an MOFV drop of 25 points (*p* < 0.001), and a reduction in unexplained IIV associated with CL/F of 19%. Two different clearance values were observed for each group, i.e., 20.4 L·h^−1^ for expressers vs. 10.1 L·h^−1^ for non-expressers. In contrast, when CYP3A4 genotypes were tested on CL/F (CYP3A4*22 carriers vs. non-carriers), no statistically significant difference was found and no reduction in the IIV associated with CL/F was observed. A cluster of CYP3A4 and CYP3A5 polymorphism was created, combining both SNPs. The different cluster phenotype groups resulted in significantly different means of C_0_ normalized by dose (Table 2), 1.23, 2.94, and 4.04 ng·mL^−1^ for HM, IM, and PM groups (*p* < 0.001), respectively. The inclusion of cluster as a covariate significantly decreased the MOFV with respect to the base model, resulting in an MOFV drop by 37.5 points (*p* < 0.001). Three different CL/F values were identified, one per each cluster phenotype, i.e., CL/F values were 19.6 L·h^−1^, 10.6 L·h^−1^, and 7.37 L·h^−1^, for high, intermediate, and poor metabolizers, respectively. After the inclusion of this covariate, a reduction of 22.5% was observed in IIV associated with CL/F.

On the other hand, the ABCB1 *C/*C SNP failed to influence the model significantly. The unexplained IIV associated with the CL/F was reduced from 49% to 38% from the base to the final covariate model. The final model parameters were estimated with good precision, the RSE% of all the parameters being lower than 35%. The eta shrinkage for CL/F in the final model was 13%. Residual unexplained variability (RUV) associated with the final model was 9.67% and the corresponding shrinkage was 23.5%. The final population pharmacokinetic parameter values are displayed in Table 3.

**Figure 2:**
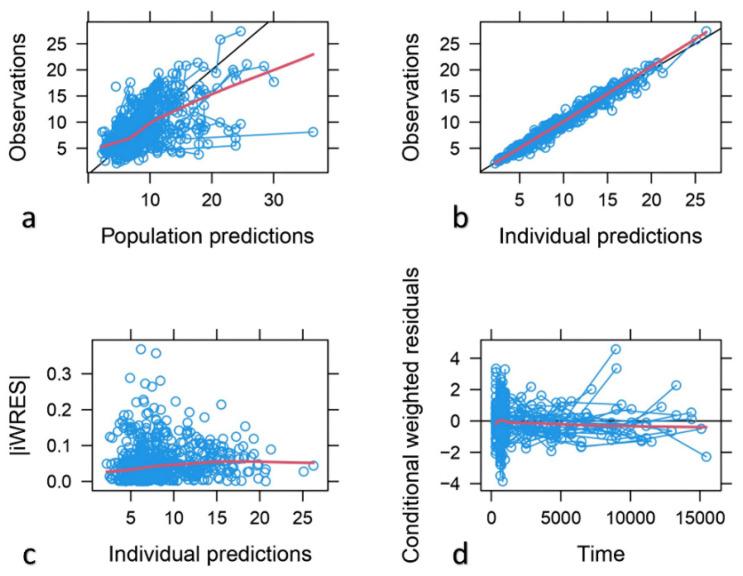
Goodness-of-fit plots of the final model. (**a**) Observed concentrations vs. population-predicted concentrations. (**b**) Observed concentrations vs. individual predicted concentrations. (**c**) Individual weighted residuals (IWRES) vs. individual predicted concentrations. (**d**) Conditional weighted residuals (CWRES) vs. time from the start of the study.

**Figure 3:**
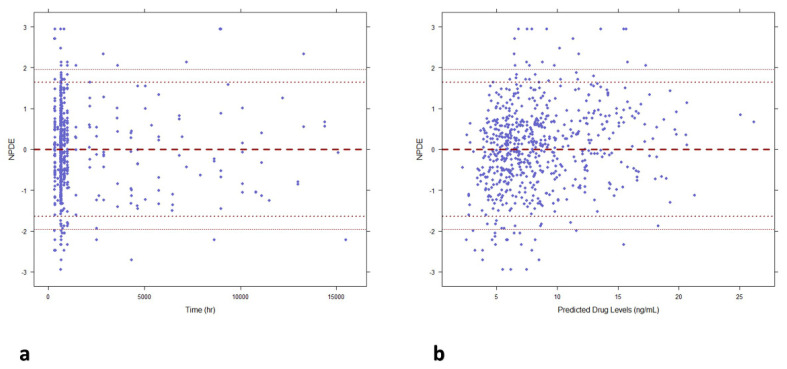
Normalized prediction distribution errors (NPDE) of the final PopPK model. (**a**) Scattered plot of NPDE vs. time from the start of the treatment. (**b**) Scattered plot of observed NPDE vs. individual predicted concentrations. The central dashed black line represents the null line, whereas the dotted and dashed black lines above and below the zero line represent the 95% confidence interval for the distribution of NPDEs.

The descriptive capability of the model was confirmed by the goodness of fit plots. Good correlations between the observed and population and individual predicted concentrations were found with a random distribution around the identity line. Individual weighted residuals (IWRES) did not show any trend when plotted against individual predicted concentrations, confirming that residual error was adequately modeled. Similarly, conditional weighted residuals (CWRES) indicated that the structural part of the model adequately described the data (Figure 2). The scatter plots of NPDE vs. time and individual predicted concentrations (Figure 3) showed a random distribution around the null line with most of the predicted NPDE values within the 95% confidence interval of the theoretical normal distribution, proving the descriptive capability of the model.

**Figure 4:**
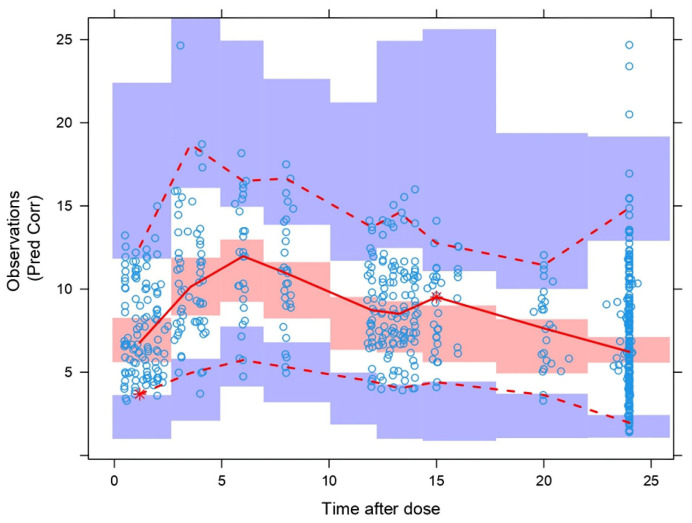
Prediction-corrected visual predictive check (pcVPC) for the final model. Tac concentrations given in ng·mL^−1^, time after dose given in hours. The solid line represents the median observed whole blood concentrations (ng·mL^−1^); prediction-corrected plasma concentration in the pcVPC to the right, and the red band represents a simulation-based 95% confidence interval for the median. The observed 5% and 95% percentiles are presented with dashed red lines, and the 95% confidence intervals for the corresponding model predicted percentiles are shown as blue bands. The observed whole blood concentrations (prediction corrected in the pcVPC) are represented by blue circles.

The pcVPC (Figure 4) showed that the model properly describes the mean tendency of the entire data. In general, the median, 5th, and 95th percentiles of the observations were within the 95% confidence intervals for the median, the 5th, and 95th percentiles of the simulated profiles.

## 4. Discussion

To the best of our knowledge, this is the first population approach study focused on the influence of genetic polymorphisms of CYP3A5, CYP3A4, and ABCB1 on Tac pharmacokinetics upon LCP-Tac administration in stable kidney transplant patients. Only in one previous study from Woillard et al. [37], a similar number of data was analyzed (637 from two full PK profiles vs. 655 in our study) from fewer patients (47 vs. 98 in our study), but no influence of genetics was explored. In another study, Henin et al. [35] included a larger set of data and also addressed the influence of CYP3A5 SNPs but not of CYP3A4 and ABCB1, which are also involved in Tac pharmacokinetics, in “de novo” patients.

Unlike previous studies in kidney transplant patients [35,37], the disposition profile of Tac after LCP-Tac administration was best described by a two-compartment model. The delayed first-order absorption was described by two transit compartments and a mean transit time of 2.91 h. The number of transit compartments had to be fixed in the final model to better estimate the central compartment distribution volume and inter-individual variability associated with this parameter. The description of the delayed absorption and disposition profile provided by our final model was consistent with that of Martial et al. [36] in stable adult hepatic transplant patients (two compartmental models, NN = 1.6 and MTT = 3.4 h). In contrast, other studies in renal transplantation [35,37] omitted one disposition compartment to better describe the complex Tac-delayed absorption from LCP-Tac, hereby characterizing two and three absorption phases with rapid/slow [37] or rapid/medium/slow [35] absorption kinetics. According to these models, the highest fraction of drug absorbed occurred in the slow (60%) [37] and the medium kinetic absorption phases (61%, MTT = 4.82 h) [35], which probably represent what occurs at the more distal parts of the small intestine as described before for LCP_Tac [46]. Thus, a scintigraphy study carried out in healthy volunteers revealed that LCP-Tac reached the colon at around 3.8–4.8 h post-administration [46]. Interestingly, our MTT (2.91 h) was between the fast (1.06 h) and medium (4.82 h) mean times reported by Henin for the absorption process. On the other hand, in our study and previous [35], in general, a high inter-individual variability was observed for parameters characterizing the absorption process. Herein lies the reason for the need for further analysis with more data/patients to confirm previous findings. In any case, all these results confirm the delayed absorption rate of Tac from LCP-Tac, with probably the highest part being absorbed in the distal part of the small intestine, but also the importance of using Erlang distribution or transit compartment models to describe it is confirmed.

Our model is less mechanistic than those previously developed [35,37], and it did not describe the multiple peaks observed in the absorption profiles (Figure 1), whereas it adequately described the mean trend of the data. The observed peak and C_0_ were correctly captured by our model as evidenced by the prediction visual predictive check plot (Figure 3). Thus, a feasible estimation of the central compartment distribution volume was found, which is crucial for a correct prediction of peak concentrations. It is of note that the total distribution volume estimated by our model (Vc/F+Vp/F) was higher than the distribution volumes previously reported by Henin (629 L vs. 452 L [35], respectively) but closer to that reported by Martial et al. [36] in hepatic transplant patients, although these authors fixed the Vp/F value at 500 L.

Among all the covariates tested, the CYP3A5 SNP expressers vs. non-expressers were statistically significant on CL/F, but no significance was found when the influence of CYP3A4 SNPs (*22 carriers vs. non-carriers) were tested on CL/F. Neither SNP associated with ABCB1 (*C carriers vs. non-carriers) showed influence on CL/F. These findings were consistent with those of the statistical analyses of exposure metrics estimated from raw data. Indeed, statistically significant differences were found in AUC/D and C_0_/D values (*p* < 0.005) when compared with CYP3A5 expressers vs. non-expressers (Table 2), with lower values for expressers that also had higher CL/F values (20.4 L·h^−1^ vs. 10.1 L·h^−1^ for expressers vs. non-expressers, respectively). In contrast, although a trend for higher exposure (AUC/D) was found for CYP3A4*22 carriers vs. non-carriers, these differences reached the level of being significant (*p* = 0.2410), but they did become significant when a higher sample size was available in the case of C_0_/D (*p* < 0.005). In any case, the relative magnitude of the differences in exposure metrics between genetic groups was lower for CYP3A4 (from 20 to 27%) than CYP3A5 (from 46 to 60%). This could be explained by a lower contribution of CYP3A4 on the Tac metabolism as a consequence of a lower intrinsic clearance [47], but also by the low number of patients of the *22 carrier group that gave rise to the mean AUC/D value estimated in this group.

As previously stated [32], all these findings led to the investigation of the effect of a combination of CYP3A4/A5 genotypes as a cluster grouped with three phenotypes (HM, IM, PM). The inclusion of the cluster on CL/F was statistically significant resulting in three different CL/F values (19.6, 10.6, and 7.37 L·h^−1^ for HM, IM, and PM, respectively) with a slightly higher reduction in MOFV. In line with this, statistically significant differences were found among AUC/D values of HM and IM or PM, but not between IM and PM. However, differences between IM and PM were evidenced when the sample size was increased in C_0_/D comparisons (*p* < 0.005). Thus, the significant inclusion of the cluster reinforced the impact of CYP3A4*22 carriers vs. non-carriers on Tac metabolism.

After comparing our results with those previously reported by our group [32], in Tac-IR formulations, similar values for HM (CL/F_HM_: 19.7, n = 47 and 19.6 L·h^−1^, n = 19 were found for Tac-IR and LCP-Tac, respectively. On the other hand, slightly higher values were found for IM (CL/F_IM_: 12.5 L·h^−1^, n = 230 and 10.6 L·h^−1^, n = 68 and PM (CL/F_PM_: 9.1 L·h^−1^, n = 27 and 7.37 L·h^−1^, n = 11) in Tac-IR vs. LCP-Tac formulations, respectively. In our previous model [32], the delayed absorption was not described with transit models, this thereby leading to lower predicted concentrations at the end of the dosing interval than expected with a transit compartment modeling. Furthermore, although differences in the inter-individual variability found in both studies can bias comparisons, a trend toward higher bioavailability (or lower CL/F values) for LCP-Tac formulation than the Tac-IR is evidenced, as should be expected [19]. Indeed, taking into account the CYP3A4/A5 expression along the gut, these differences could be attributed to the different release profiles of Tac from both formulations. Unlike Tac-IR, according to the results of our model, LCP-Tac is not immediately released at the proximal part of the intestine, where due to the higher expression of CYP3A4/A5, a higher first-pass effect takes place. Regarding the effect of ABCB1 SNP (*T/*T, *C/*T, and *C/*C SNPs), no influence on CL/F was detected, nor differences between exposure metrics of ABCB1*C vs. non-carriers (Table 2). This result is in agreement with previous studies carried out with the Tac-IR formulation exposure [7,25].

Our CL/F values were lower in CYP3A5 expressers vs. non-expressers (20.4 L·h^−1^ and 10.1 L·h^−1^, respectively) than those reported by Henin et al. [35] in “de novo” patients. Specifically in this population, considering a mean bodyweight of around 70 kg, CL/F of 33.2 and 20 L·h^−1^ were estimated for CYP3A5 expressers. These differences could be due to co-administered corticosteroids at high doses in the early period post-transplant but also to hematocrit concentrations between “de novo” and stable patient sub-populations. Patients of our study received reduced doses or discontinued corticosteroids, compared to the higher doses given to “de novo” patients, in accordance with standard practice. Higher doses of corticosteroids can lead to greater induction of the CYP3A4 metabolizing enzyme and thus lead to higher Tac clearance [48]. This finding again supports the role of CYP3A4 on the metabolism of Tac. On the other hand, hematocrit concentrations of our study were higher (from 37.4 to 44.0% (IQR)) than those reported in “de novo” patients [35] (from 22.9 to 37%). As a restrictive clearance drug, Tac is expected to decrease its clearance as the free fraction decreases, as occurs in our stable patients where hematocrit concentrations reach its almost normal values compared with “de novo” patients. This was also the reason why probably this covariate was not predictive of CL/F variability in the final model. All these findings support the development of the current model for LCP-Tac in stable patients.

However, this study has some limitations. It was performed in stable kidney transplant recipients more than 6 months after transplantation. Therefore, it may not be appropriate for de novo transplant recipients receiving LCP-Tac. Another limitation is the low prevalence of PM patients in the Caucasian population. Further studies with a larger sample size are required to improve the model and confirm the current results regarding the influence of CYP3A4*1 and CYP3A5*3 genetic polymorphisms on Tac exposure in stable kidney transplant patients treated with LCP-Tac. Finally, external validation of this PopPK model would be needed before using the final model as a support tool during therapeutic drug monitoring.

## 5. Conclusions

A new population pharmacokinetic model has been developed in stable renal adult transplant patients upon LCP-Tac administration. The influence of CYP3A4/A5 SNPs as a combined cluster including three different phenotypes has been identified as a powerful covariate to describe part of the inter-individual variability associated with the apparent elimination clearance. This suggests the inclusion of this covariate could improve dose optimization in routine clinical practice.

## Figures and Tables

**Table 1:** Demographic, biochemical, and clinical characteristics of the patients included in the study.

Characteristics	All Patients	Full PK Profile	Sparse Sampling (C_0_)
No. of Patients	98	30	68
No. of Sampling	655	480	175
Gender (Male/Female)	68/30	20/10	45/23
Weight (kg)	74.73 (65–81.13)	72.82 (63.75–80)	75.36 (65.5–88)
Age (Years)	56 (46–68)	57 (48–67)	56 (45–68)
BMI (kg·m^−2^)	26.33 (22.94–28.94)	26.44 (23.44–29.70)	26.28 (22.89–28.64)
HTC (%)	40.53 (37.4–44.0)	39.7 (37.1–41.9)	40.64 (37.42–44.0)
GF (mL·min^−1^)	47.72 (36–58)	54.86 (45.5–67)	46.7 (36–57)
CR (μmol·L^−1^)	146.9 (116–163)	130.47 (106.5–149)	149 (118–164)
CYP3A5 Genotype			
*1/*1	4 (4.1%)	1 (3.3%)	3 (4.5%)
*1/*3	17 (17.3%)	9 (30%)	8 (11.5%)
*3/*3	77 (78.6%)	20 (66.7%)	57 (84%)
CYP3A4 Genotype			
*1/*1	86 (86.7%)	28 (93.3%)	58 (84%)
*1/*22	12 (13.3%)	2 (6.7%)	10(16%)
Cluster			
*HM*	19 (19.4%)	10 (33%)	9 (13.25%)
*IM*	68 (69.4%)	18 (60%)	50 (73.5%)
*PM*	11 (11.2%)	2 (7%)	9 (13.25%)
ABCB1 Genotype			
*T/*T	21 (21%)	9 (30%)	12 (18%)
*C/*T	46 (47%)	12 (40%)	34 (50%)
*C/*C	31 (32%)	9 (30%)	22 (32%)

Values are given as arithmetic mean (interquartile range) for continuous variables, and as count (percentage) for categorical variables; BMI, body mass index; HTC, hematocrit; GF, glomerular filtration rate estimated by the CKD-EPI formula; Cr, serum creatinine; HM, high metabolizer; IM, intermediate metabolizer; PM, poor metabolizer.

**Table 2:** Comparative normalized-by-dose C_0_ and AUC values sorted by the CYP3A5, CYP3A4, and ABCB1 SNP groups and by the three different cluster phenotypes.

Genotype Group	Dose (mg·day^−1^)	C_0_	N	AUC	N	C_0_/D	*p*-Value *	AUC/D	*p*-Value z
		(ng·mL^−1^)		(ng·h·mL^−1^)					
CYP3A5									
CYP3A5 *1/*1, *1/*3	5 (2–12)	6.14 (5.47–6.90)	46	252 (180–353)	10	1.22 (1.07–1.42)	<0.001	51 (41–64)	<0.001
CYP3A5 *3/*3	2 (0.5–8)	6.4 (6.06–6.75)	187	191 (165–223)	20	3.08 (2.94–3.44)		95 (81–110)	
CYP3A4									
CYP3A4 *1/*1	2.5 (0.75–12)	6.19 (5.86–6.54)	180	207 (178–241)	28	2.42 (2.23–2.67)	<0.05	76 (64–91)	0.2410
CYP3A4 *1/*22	1.75 (0.5–8)	7.05 (5.71–7.20)	53	198 (99–398)	2	3.01 (2.80–4.15)		122 (76–194)	
CLUSTER									
High-Metabolizer	3 (2–12)	6.16 (5.47–6.93)	46	252 (180–353)	10	1.23 (1.06–1.42)	<0.001 #	51 (41–64)	<0.001 #
							<0.001 &		<0.05 &
Intermediate-Metabolizer	2 (0.75–8)	6.16 (5.80–6.54)	141	190 (161–224)	18	2.91 (2.67–3.18)	<0.05 $	92 (80–106)	0.5756 $
Poor-Metabolizer	1.75 (0.5–8)	7.3 (6.60–8.10)	46	198 (99–398)	2	4.04 (0.48–4.1)		122 (76–194)	
ABCB1									
ABCB1 *C/*T, *C/*C	2 (0.5–12)	6.25 (5.94–6.63)	181	203 (170–242)	21	2.58 (2.33–2.85)	0.3073	78 (64–95)	0.4537
ABCB1 *T/*T	2.125 (1–8)	6.4 (5.79–7.30)	52	213 (163–279)	9	2.81 (2.48–3.19)		83 (62–112)	

Values are reported as geometric means (95% CI). Abbreviations: AUC, area under the whole-blood concentration-time curve; Ctrough: trough whole blood concentrations, Ctrough/D and AUC/D normalized by dose Ctrough and AUC values. N = number of occasions data analyzed is the same for Ctrough and Ctrough/D. Similarly, N is the same for AUC and AUC/D. Dose expressed as median (range). * *p*-values for Ctrough/D mean values statistical comparisons, z *p*-values for AUC/D mean values statistical comparisons, # differences between HM and IM, & differences between HM and PM, $ differences between IM and PM.

**Table 3:** Pharmacokinetic parameter estimates of the final model. Relative standard errors are given in parenthesis.

Parameter	Value (RSE %)	IIV % (RSE %)
CL/F_HM_ (L·h^−1^)	19.6 (10)	37.9 (17.9)
CL/F_IM_ (L·h^−1^)	10.6 (5.2)	-
CL/F_PM_ (L·h^−1^)	7.37 (11.9)	-
Vc/F (L)	169 (17.2)	70 (41.4)
CL_D_/F (L·h^−1^)	37.6 (13.5)	-
Ka (h^−1^)	0.72 (33.2)	-
Vp (L)	460 (27.8)	75 (44.3)
MTT (h)	2.91 (15.5)	54.6 (37.1)
NN	2 FIX	-
IOV (%)	44.8 (27.4)	-
RE (%)	9.67 (8)	-

RSE: relative standard error; IIV: inter-individual variability; CL/F_HM_: elimination clearance (high metabolizer); CL/F_IM_ (L·h^−1^): elimination clearance (intermediate metabolizer); CL/F_PM_ (L·h^−1^): elimination clearance (poor metabolizer); Vc/F (L): central compartment distribution volume; CL_D_/F (L·h^−1^): distributional clearance; Ka (h^−1^): absorption rate constant; Vp (L): peripheral compartment distribution volume; MTT (h): mean transit time; NN: number of transit compartments; IOV: inter-occasion variability; RE: residual error.

## Data Availability

Data available on request due to restrictions eg privacy or ethical.

## References

[1] Bowman L.J., Brennan D.C. (2008). The role of tacrolimus in renal transplantation. Expert Opin. Pharmacother..

[2] Kamel M., Kadian M., Srinivas T., Taber D., Salas M.A.P. (2016). Tacrolimus confers lower acute rejection rates and better renal allograft survival compared to cyclosporine. World J. Transplant..

[3] Venkataramanan R., Swaminathan A., Prasad T., Jain A., Zuckerman S., Warty V., McMichael J., Lever J., Burckart G., Starzl T. (1995). Clinical Pharmacokinetics of Tacrolimus. Clin. Pharmacokinet..

[4] Staatz C.E., Tett S.E. (2004). Clinical pharmacokinetics and pharmacodynamics of tacrolimus in solid organ transplantation. Clin. Pharmacokinet..

[5] Kuypers D.R.J. (2020). Intrapatient Variability of Tacrolimus Exposure in Solid Organ Transplantation: A Novel Marker for Clinical Outcome. Clin. Pharmacol. Ther..

[6] Neylan J.F. (1998). Effect of race and immunosuppression in renal transplantation: Three- year survival results from a US multicenter, randomized trial. Transplant. Proc..

[7] Staatz C.E., Goodman L.K., Tett S.E. (2010). Effect of CYP3A and ABCB1 single nucleotide polymorphisms on the pharmacokinetics and pharmacodynamics of calcineurin inhibitors: Part I. Clin. Pharmacokinet..

[8] Bekersky I., Dressler D., Mekki Q.A. (2001). Effect of low- and high-fat meals on tacrolimus absorption following 5 mg single oral doses to healthy human subjects. J. Clin. Pharmacol..

[9] Tuteja S., Alloway R.R., Johnson J.A., Gaber A.O. (2001). The effect of gut metabolism on tacrolimus bioavailability in renal transplant recipients. Transplantation.

[10] Han S.S., Kim D.H., Lee S.M., Han N.Y., Oh J.M., Ha J., Kim Y.S. (2012). Pharmacokinetics of tacrolimus according to body composition in recipients of kidney transplants. Kidney Res. Clin. Pract..

[11] Seegars M.B., Tooze J., Isom S., Anders B., Kennedy L., Rodriguez C. (2018). Tacrolimus Levels and Correlation to Age and Weight Calculation. Biol. Blood Marrow Transplant..

[12] Katari S.R., Magnone M., Shapiro R., Jordan M., Scantlebury V., Vivas C., Gritsch A., Mccauley J., Starzl T., Demetris A.J. (1997). Clinical features of acute reversible tacrolimus (FK 506) nephrotoxicity in kidney transplant recipients. Clin. Transplant..

[13] Weng L.C., Chiang Y.J., Lin M.H., Hsieh C.Y., Lin S.C., Wei T.Y., Chou H.F. (2014). Association Between Use of FK506 and Prevalence of Post-transplantation Diabetes Mellitus in Kidney Transplant Patients. Transplant. Proc..

[14] Sierra-Hidalgo F., Martínez-Salio A., Moreno-García S., De Pablo-Fernández E., Correas-Callero E., Ruiz-Morales J. (2009). Akinetic mutism induced by tacrolimus. Clin. Neuropharmacol..

[15] Wallemacq P.E., Furlan V., Möller A., Schäfer A., Stadler P., Firdaous I., Taburet A.M., Reding R., Clement De Clety S., De Ville De Goyet J. (1998). Pharmacokinetics of tacrolimus (FK506) in paediatric liver transplant recipients. Eur. J. Drug Metab. Pharmacokinet..

[16] Tremblay S., Nigro V., Weinberg J., Woodle E.S., Alloway R.R. (2017). A Steady-State Head-to-Head Pharmacokinetic Comparison of All FK-506 (Tacrolimus) Formulations (ASTCOFF): An Open-Label, Prospective, Randomized, Two-Arm, Three-Period Crossover Study. Am. J. Transplant..

[17] Gaber A.O., Alloway R.R., Bodziak K., Kaplan B., Bunnapradist S. (2013). Conversion from twice-daily tacrolimus capsules to once-daily extended-release tacrolimus (LCPT): A phase 2 trial of stable renal transplant recipients. Transplantation.

[18] Trofe-Clark J., Brennan D.C., West-Thielke P., Milone M.C., Lim M.A., Neubauer R., Nigro V., Bloom R.D. (2018). Results of ASERTAA, a Randomized Prospective Crossover Pharmacogenetic Study of Immediate-Release Versus Extended-Release Tacrolimus in African American Kidney Transplant Recipients. Am. J. Kidney Dis..

[19] Staatz C.E., Tett S.E. (2015). Clinical Pharmacokinetics of Once-Daily Tacrolimus in Solid-Organ Transplant Patients. Clin. Pharmacokinet..

[20] Budde K., Bunnapradist S., Grinyo J.M., Ciechanowski K., Denny J.E., Silva H.T., Rostaing L. (2014). Novel Once-Daily Extended-Release Tacrolimus (LCPT) Versus Twice-Daily Tacrolimus in De Novo Kidney Transplants: One-Year Results of Phase III, Double-Blind, Randomized Trial. Am. J. Transplant..

[21] Thörn M., Finnström N., Lundgren S., Rane A., Lööf L. (2005). Cytochromes P450 and MDR1 mRNA expression along the human gastrointestinal tract. Br. J. Clin. Pharmacol..

[22] Birdwell K.A., Decker B., Barbarino J.M., Peterson J.F., Stein C.M., Sadee W., Wang D., Vinks A.A., He Y., Swen J.J. (2015). Clinical Pharmacogenetics Implementation Consortium (CPIC) Guidelines for CYP3A5 Genotype and Tacrolimus Dosing. Clin. Pharmacol. Ther..

[23] Zhang X., Liu Z.H., Zheng J.M., Chen Z.H., Tang Z., Chen J.S., Li L.S. (2005). Influence of CYP3A5 and MDR1 polymorphisms on tacrolimus concentration in the early stage after renal transplantation. Clin. Transplant..

[24] De Jonge H., De Loor H., Verbeke K., Vanrenterghem Y., Kuypers D.R. (2012). In vivo CYP3A4 activity, CYP3A5 genotype, and hematocrit predict tacrolimus dose requirements and clearance in renal transplant patients. Clin. Pharmacol. Ther..

[25] Capron A., Mourad M., De Meyer M., De Pauw L., Eddour D.C., Latinne D., Elens L., Haufroid V., Wallemacq P. (2010). CYP3A5 and ABCB1 polymorphisms influence tacrolimus concentrations in peripheral blood mononuclear cells after renal transplantation. Pharmacogenomics.

[26] Pallet N., Jannot A.S., El Bahri M., Etienne I., Buchler M., De Ligny B.H., Choukroun G., Colosio C., Thierry A., Vigneau C. (2015). Kidney Transplant Recipients Carrying the *CYP3A4*22* Allelic Variant Have Reduced Tacrolimus Clearance and Often Reach Supratherapeutic Tacrolimus Concentrations. Am. J. Transplant..

[27] Abdel-Kahaar E., Winter S., Tremmel R., Schaeffeler E., Olbricht C.J., Wieland E., Schwab M., Shipkova M., Jaeger S.U. (2019). The impact of *CYP3A4*22* on tacrolimus pharmacokinetics and outcome in clinical practice at a single kidney transplant center. Front. Genet..

[28] Tang J.T., Andrews L.M., Van Gelder T., Shi Y.Y., Van Schaik R.H.N., Wang L.L., Hesselink D.A. (2016). Pharmacogenetic aspects of the use of tacrolimus in renal transplantation: Recent developments and ethnic considerations. Expert Opin. Drug Metab. Toxicol..

[29] Brooks E., Tett S.E., Isbel N.M., Staatz C.E. (2016). Population Pharmacokinetic Modelling and Bayesian Estimation of Tacrolimus Exposure: Is this Clinically Useful for Dosage Prediction Yet?. Clin. Pharmacokinet..

[30] Woillard J.B., Mourad M., Neely M., Capron A., van Schaik R.H., van Gelder T., Lloberas N., Hesselink D.A., Marquet P., Haufroid V. (2017). Tacrolimus updated guidelines through popPK modeling: How to benefit more from CYP3A pre-emptive genotyping prior to kidney transplantation. Front. Pharmacol..

[31] Andrews L.M., Hesselink D.A., van Schaik R.H.N., van Gelder T., de Fijter J.W., Lloberas N., Elens L., Moes D.J.A.R., de Winter B.C.M. (2019). A population pharmacokinetic model to predict the individual starting dose of tacrolimus in adult renal transplant recipients. Br. J. Clin. Pharmacol..

[32] Andreu F., Colom H., Elens L., van Gelder T., van Schaik R.H.N., Hesselink D.A., Bestard O., Torras J., Cruzado J.M., Grinyó J.M. (2017). A New *CYP3A5*3* and *CYP3A4*22* Cluster Influencing Tacrolimus Target Concentrations: A Population Approach. Clin. Pharmacokinet..

[33] Andreu F., Colom H., Grinyó J.M., Torras J., Cruzado J.M., Lloberas N. (2015). Development of a population PK model of tacrolimus for adaptive dosage control in stable kidney transplant patients. Ther. Drug Monit..

[34] Woillard J.B., Saint-Marcoux F., Debord J., Åsberg A. (2018). Pharmacokinetic models to assist the prescriber in choosing the best tacrolimus dose. Pharmacol. Res..

[35] Henin E., Govoni M., Cella M., Laveille C., Piotti G. (2021). Therapeutic Drug Monitoring Strategies for Envarsus in De Novo Kidney Transplant Patients Using Population Modelling and Simulations. Adv. Ther..

[36] Martial L.C., Biewenga M., Ruijter B.N., Keizer R., Swen J.J., van Hoek B., Moes D.J.A.R. (2021). Population pharmacokinetics and genetics of oral meltdose tacrolimus (Envarsus) in stable adult liver transplant recipients. Br. J. Clin. Pharmacol..

[37] Woillard J.B., Debord J., Monchaud C., Saint-Marcoux F., Marquet P. (2017). Population Pharmacokinetics and Bayesian Estimators for Refined Dose Adjustment of a New Tacrolimus Formulation in Kidney and Liver Transplant Patients. Clin. Pharmacokinet..

[38] Rigo-Bonnin R., Arbiol-Roca A., de Aledo-Castillo J.M.G., Alía P. (2015). Simultaneous Measurement of Cyclosporine A, Everolimus, Sirolimus and Tacrolimus Concentrations in Human Blood by UPLC–MS/MS. Chromatographia.

[39] Savic R.M., Jonker D.M., Kerbusch T., Karlsson M.O. (2007). Implementation of a transit compartment model for describing drug absorption in pharmacokinetic studies. J. Pharmacokinet. Pharmacodyn..

[40] Karlsson M.O., Sheiner L.B. (1993). The importance of modeling interoccasion variability in population pharmacokinetic analyses. J. Pharmacokinet. Biopharm..

[41] Yamaoka K., Nakagawa T., Uno T. (1978). Application of Akaike’s information criterion (AIC) in the evaluation of linear pharmacokinetic equations. J. Pharmacokinet. Biopharm..

[42] Savic R.M., Karlsson M.O. (2009). Importance of Shrinkage in Empirical Bayes Estimates for Diagnostics: Problems and Solutions. AAPS J..

[43] Jonsson E.N., Karlsson M.O. (1998). Automated covariate model building within NONMEM. Pharm. Res..

[44] Bergstrand M., Hooker A.C., Wallin J.E., Karlsson M.O. (2011). Prediction-corrected visual predictive checks for diagnosing nonlinear mixed-effects models. AAPS J..

[45] Comets E., Brendel K., Mentré F. (2008). Computing normalised prediction distribution errors to evaluate nonlinear mixed-effect models: The npde add-on package for R. Comput. Methods Programs Biomed..

[46] Nigro V., Glicklich A., Weinberg J. (2013). Improved Bioavailability of MELTDOSE Once-Daily Formulation of Tacrolimus (LCP-Tacro) with Controlled Agglomeration Allows for Consistent Absorption over 24 Hrs: A Scintigraphic and Pharmacokinetic Evaluation. Am. J. Transplant..

[47] Kamdem L.K., Streit F., Zanger U.M., Brockmöller J., Oellerich M., Armstrong V.W., Wojnowski L. (2005). Contribution of CYP3A5 to the in vitro hepatic clearance of tacrolimus. Clin. Chem..

[48] Undre N.A. (2003). Pharmacokinetics of tacrolimus-based combination therapies. Nephrol. Dial. Transplant..

